# An Exploratory Study on the Interrelation of Breast Cancer Molecular Phenotypes with Breast Cancer-Associated Adipose Tissues (BCAATs), Neoadjuvant, and Adjuvant Therapies: A Focus on Prognosis and Survival

**DOI:** 10.3390/medsci14030403

**Published:** 2026-07-18

**Authors:** Mihaela Maria Pasca Fenesan, Razvan George Bogdan, Andrei Alexandru Cosma, Vlad Vornicu, Eugen Melnic, Diana Veronica Radu, Patricia Baran, Zorin Crainiceanu, Rodica Elena Heredea, Olga Cernetchi, Anca Maria Cimpean

**Affiliations:** 1 Doctoral School, “Victor Babes” University of Medicine and Pharmacy, 300041 Timisoara, Romania; mihaela.fenesan@umft.ro (M.M.P.F.); razvan.bogdan@umft.ro (R.G.B.); vlad.vornicu@umft.ro (V.V.); berzava.patricia@umft.ro (P.B.); 2 Oncohelp Clinic, 300425 Timisoara, Romania; cosma.andrei@umft.ro; 3 Department of Microscopic Morphology/Histology, “Victor Babes” University of Medicine and Pharmacy, 300041 Timisoara, Romania; 4 Plastic Surgery Department, “Victor Babes” University of Medicine and Pharmacy, 300041 Timisoara, Romania; crainiceanu.zorin@umft.ro; 5 Department of Pathology, Nicolae Testemitanu State University of Medicine and Pharmacy, 2300 Chisinau, Moldova; 6 Food Technologies Department, University of Life Sciences King Mihai I, 300645 Timisoara, Romania; dianadogaru@usvt.ro; 7 Discipline of Clinical Practical Skills, Department I Nursing, Faculty of Medicine, “Victor Babes” University of Medicine and Pharmacy, 300041 Timișoara, Romania; elena-rodica.heredea@umft.ro; 8 Department of Obstetrics and Gynecology, Nicolae Testemitanu State University of Medicine and Pharmacy, 2300 Chisinau, Moldova; olga.cernetchi@usmf.md; 9 Center of Genomic Medicine, “Victor Babes” University of Medicine and Pharmacy, 300041 Timisoara, Romania; 10 Research Center for Pharmaco-Toxicological Evaluation, “Victor Babes” University of Medicine and Pharmacy, 300041 Timisoara, Romania; 11 Center of Expertise for Rare Vascular Disease in Children, Emergency Hospital for Children Louis Turcanu, 300011 Timisoara, Romania

**Keywords:** breast cancer (BC), breast cancer-associated adipose tissue (BCAAT), body mass index (BMI), neoadjuvant therapy, adjuvant therapy, survival, prognosis

## Abstract

Background/Aim. We previously described four distinct breast cancer (BC)-associated adipose tissue (BCAAT) subtypes that have a significant impact on survival. In this exploratory study, we aimed to determine whether these BCAAT subtypes exhibit significant correlations with neoadjuvant and adjuvant therapies and BC molecular subtypes. Methods. Four BCAAT subtypes previously identified as fibroblast-rich (F^Rich^_BCAAT), myofibroblast-rich (MyoF^Rich^_BCAAT), vascular-rich (V^Rich^_BCAAT), and mixed vascular- and inflammation-rich (VI^Rich^_BCAAT) were analyzed according to their distribution in BC molecular subtypes, as well as in relation to neoadjuvant therapy and survival. Results. Triple-negative BC and Luminal B (LB) BC are related to V^Rich^ and VI^Rich^ BCAAT subtypes and were affected by Epirubicin/Cyclophosphamide (EC)+ paclitaxel (PTX) therapy, which significantly enhanced overall survival (OS) and disease-free survival (DFS). For the Luminal A (LA) BC subtype, EC + docetaxel (DTX) had significant impact on survival independent of BCAAT subtype. Conclusions. BCAAT subtypes strongly influenced neoadjuvant therapy response and survival depending on BC molecular subtype.

## 1. Introduction

Breast cancer is now understood as a multifaceted condition rather than merely an uncontrolled lump [1]. Worldwide, 2.3 million new cases are established each year, one every fourteen seconds on average. Although death rates have decreased, the incidence is rising by 0.5% yearly in high-income nations due to more precise, targeted treatments and earlier screening procedures. Geography significantly affects health outcomes: the five-year survival rate in rural sub-Saharan Africa is 40% compared to 66% in India and around 90% in Sweden [2,3]. In contrast to the decline in mortality rates in 29 nations with exceptionally high Human Development Index (HDI) scores, low-HDI nations will bear the brunt of the crisis by 2050, with new cases increasing by 38% and fatalities by 68%. Addressing disparities and monitoring cancer reduction targets requires high-quality vital status and cancer data, as well as ongoing improvements in early diagnosis and treatment availability in countries with low and medium HDI scores [2].

Several recent studies on BC identified genomic instability as a main factor in the lack of or partial response to neoadjuvant/adjuvant therapies [4,5]. Few studies have examined microscopic changes related to neoadjuvant/adjuvant therapy in breast cancer [6,7,8]. Zhou et al. [9] developed an artificial intelligence-based model to predict the response to neoadjuvant therapy. For this purpose, hematoxylin and eosin staining was used alongside inflammatory microenvironment evaluation including macrophages, mast cells, and tumor-infiltrating lymphocytes [9]. Other microscopic data used to assess neoadjuvant therapy efficiency included lymph node evaluation results [10], tissue microenvironment structural components such as matrix metalloproteinases [11], desmoplastic reactions [12], and adipose tissue morphologic and molecular remodeling [13,14,15]. There is strong evidence for the role of adipose tissue in the development and progression of BC, but some authors have only evaluated its role in association with patients’ obesity status [16,17,18,19,20]. Few authors have linked obesity to immunohistochemically determined BC molecular subtypes [19,21] or to the impact on neoadjuvant/adjuvant therapy [22,23,24]. Fewer have focused on peritumoral adipose tissue inflammation and/or vascular components [25,26,27]. Although there is little interest in the microscopic and molecular features of BC peritumoral adipose tissue, recent data suggest that tissular components of the adipose tissue strongly influence therapy response. In the recently updated “Hallmarks of Cancer” paper [28], Hannahan included obesity and peritumor adipose microenvironment as crucial factors in the development, prognosis, and survival of malignant tumors. The authors also suggested that obesity may strongly influence the response to therapy [28].

Recently, based on double immunostaining, we described four different microscopic subtypes of BC-associated adipose tissue and found that they have predominant effects according to age and BMI, in addition to influencing survival [29]. The impact of each BCAAT microscopic subtype on neoadjuvant and adjuvant therapy has not been evaluated before. Dulgar et al. [30] reported that there is a correlation between adipose tissue metabolic activity, body mass index (BMI), and chemotherapeutic efficacy. Poor pathological response to neoadjuvant therapy and a high body mass index were both linked to a low visceral/subcutaneous fat ratio. To better anticipate the reaction to neoadjuvant therapy, the visceral-to-subcutaneous fat ratio may be a helpful indicator. Obesity is strongly associated with decreased neoadjuvant therapy efficacy, according to epidemiological studies [31]. Mutual interplay between cancer cells and adipocytes, which are the predominant stromal component of breast tissue, has been observed [32]. Through the provision of fatty acids (FAs), the enhancement of lipid oxidation, and the promotion of stem-like characteristics, cancer-associated adipocytes (CAAs) contribute to the development of chemoresistance [33,34,35]. A chemoresistant phenotype is supported by the activation of STAT3 and carnitine palmitoyltransferase 1B, which is mediated by leptin [36]. Adipose stromal cells in obese persons adopt pro-tumorigenic phenotypes, which contribute to resistance [37]. These phenotypes include myofibroblast proliferation in obese individuals and inflammatory proliferation in lean individuals.

All the data presented above indirectly suggest that peritumor adipose tissue impacts the response to neoadjuvant/adjuvant therapy. Less data are available related to the cellular and vascular heterogeneity of peritumor adipose tissue, molecular subtypes, and neoadjuvant/adjuvant therapy response. We aimed to determine if the BCAAT subtypes described previously by our team, together with BMI, age, tertiary lymphoid structures (TLSs), and survival, are significantly correlated to neoadjuvant/adjuvant therapies in molecular subtypes of breast cancer.

## 2. Materials and Methods

### 2.1. Ethical Considerations in Selecting Patients and Related Selection Criteria

This was an exploratory retrospective study which initially included 109 women, aged 32 to 79, who underwent histological investigation and received a diagnosis of ductal invasive carcinoma between 2015 and 2022. Tissues were preserved as formalin-fixed paraffin-embedded (FFPE) samples. Two separate pathologists further analyzed the FFPE samples to confirm the diagnosis and identify which cases were appropriate for immunohistochemistry (IHC). For each patient, a molecular profile based on immunohistochemical markers was created. The markers included the proliferative index (Ki67), hormone receptors (ER, PR), and human epidermal growth factor receptor 2 (HER2). Without this, it would be impossible to comprehend the different molecular subtypes of breast cancer. To accomplish our goals, we selected 53 people whose clinical, histological, and treatment profiles were deemed relevant for this study. The relatively small number of cases, which (n = 53) is enough for an exploratory study, was the result of several reasons strongly related to (i) the small geographic area relative to the number of hospitals (western Romania is a relatively small area with several oncology hospitals, where patients’ homes are dispersed between hospitals, and few of the hospitals are university hospitals that allow research activity). (ii) Fifty-three out of one hundred and nine total BC cases had proper paraffin block samples (before starting the study, as we mentioned above, the quality of formalin-fixed paraffin-embedded (FFPE) blocks and sections was reevaluated by two pathologists in relation to several microscopic criteria, followed by the selection of specimens suitable for immunohistochemistry (IHC)); (iii) technical issues related to IHC techniques such as the loss of tissue sections during preprocessing, technical issues related to the automated program of the autostainer, and IHC antibody sensitivity and specificity). When the reassessment of BC molecular subtypes yielded controversial results compared to the initial evaluation, these cases were excluded from the study.

In this study, we examine key clinicopathologic and therapeutic factors, such as age, body mass index, lympho-vascular invasion, perineural invasion, recurrence, and survival. The Victor Babeș University of Medicine and Pharmacy in Timișoara’s Research Ethics Council (No. 49/28.09.2018) investigated and approved the use of a standardized form to obtain each patient’s informed permission.

### 2.2. Objectives

The main objective of our study was to determine if the patients included responded differently to neoadjuvant/adjuvant therapy related to the BCAAT subtypes previously defined by our group. We focused on identifying factors influencing neoadjuvant therapy choice and their impact on the future survival and prognosis of BC patients.

Additional objectives included the potential impact of BCAAT subtypes on other clinical–pathological and prognostic parameters such as survival, body mass index (BMI), and age or other tumor microenvironment components, such as inflammatory cells organized as tertiary lymphoid structures (TLSs).

### 2.3. Inclusion and Exclusion Criteria

We collected data from breast cancer patients who met all of the following criteria: a full pathologic profile of the disease (TNM staging, grade, IHC surrogate markers for molecular classification, neoadjuvant and adjuvant therapy protocol), BMI, the presence of tumor lymph nodes (TLSs), whether the patients were pre- or postmenopausal, informed consent, and permission to use their biopsies for research.

Patients who did not meet the inclusion criteria were those under the age of eighteen, who had not provided informed consent, who had diabetes or another metabolic disease, who had an inflammatory condition such as an autoimmune disease, who had a cardiovascular disease, or who had renal failure that was documented before or during the diagnosis of breast cancer.

### 2.4. Selected Patients’ Characteristics

We summarized the patients’ data related to age, BCAAT subtype, BC molecular subtypes assessed before neoadjuvant therapy, months of survival, and neoadjuvant therapy protocol. All these data are presented in Table 1. BC molecular subtypes were established by using the last classification related to molecular subtypes.

### 2.5. Statistical Data Analysis

JAMOVI (The jamovi Project, Sydney, Australia, latest version 2.7.37) on macOS was used for statistical analysis. We performed a survival rate analysis using the Kaplan–Meier survival test. If the *p*-value was less than 0.05, we considered it a statistically significant correlation. We performed statistical analysis for all BC molecular subtypes except for the HER2 subgroup due to an extremely low number of cases (n = 3). The group with the next lowest number of cases was LB-HER2 (n = 7); statistical analysis was performed for this subgroup, but additional correction tests used for small sample size cohorts strongly suggested that the results obtained for this LB-HER2 subgroup were questionable. Therefore, we report the results for the LB-HER2 subgroup briefly and critically at the end of the Section 3 without putting forth any hypothesis derived from this statistical analysis.

Multivariate analysis of covariance was performed using MANCOVA tests in JAMOVI statistical software. A *p*-value of less than 0.05 was considered statistically significant.

## 3. Results

The molecular classification of BC specimens was carried out by interpreting surrogate markers for estrogen receptors (ERs), progesterone receptor (PRs), the proliferation index (Ki67), and HER 2 expression. We reported BC molecular subtypes according to the BCAAT subgroups recently described by our team [29].

**Figure 1:**
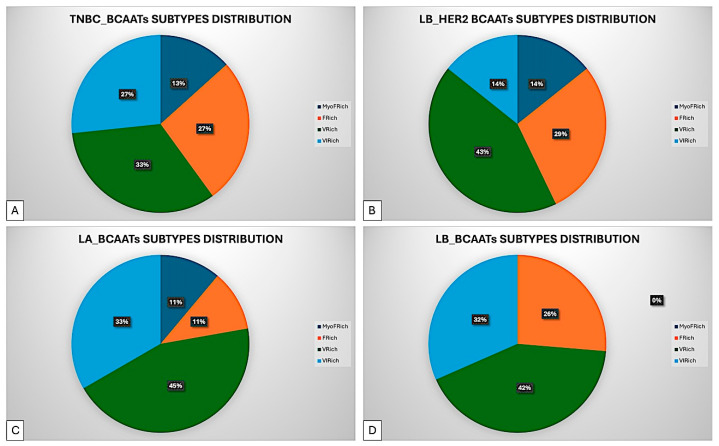
Comparative analysis of BCAAT subtype distribution across several BC molecular subtypes: TNBC (**A**), LB_HER2 (**B**), LA (**C**), and LB (**D**). Note that LB_BCAAT subtype had no MyoF^Rich^ adipose tissue subtype (**D**) and the LA subtype had the lowest percentage of F^Rich^ BCAAT subtype (**C**). Also, TNBC and LB_HER2 subtypes had the highest percentage of F^Rich^ BCAAT subtype (**A**,**B**).

Comparative analysis of BCAAT distribution across the BC molecular subtypes is summarized in Figure 1.

**Figure 2:**
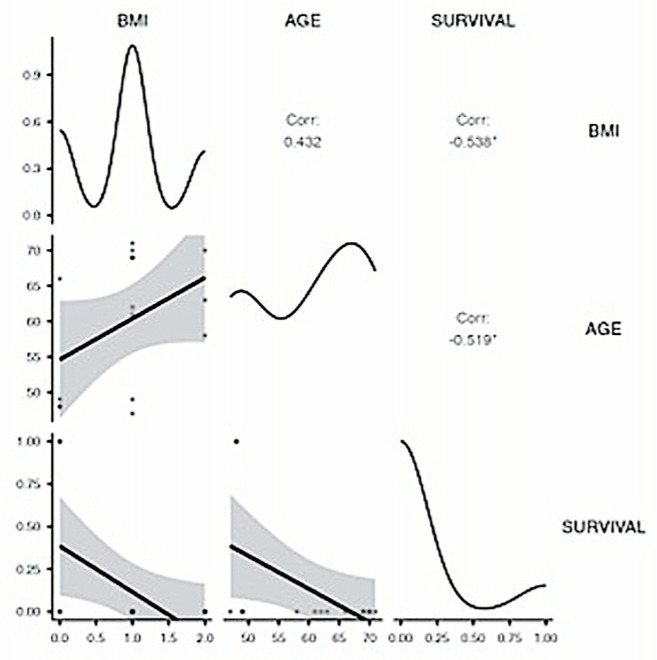
Correlation plot for TNBC cases related to survival and BMI. Thick lines represent the best-fit regression line for the relationship between the two variables being compared (e.g., BMI vs. age). Thin lines (or the shaded gray area) represent the confidence interval around the regression line, usually showing the region within which the true regression line is likely to be found (commonly 95%). The variable listed at the bottom of the column is on the x-axis. The variable listed at the left of the row is on the y-axis. Significant correlation for * *p* < 0.05, ** *p* < 0.01, and *** *p* < 0.001; one-tailed.

Of all cases analyzed, 28.3% had no expression of ER, PR, and/or HER2 and were classified as triple-negative breast cancer (TNBC). In the group of TNBC cases, 33% were included in the V^Rich^ BCAAT subtype, 27% in the VI^Rich^ BCAAT subtype, 27% in the F^Rich^ BCAAT subtype, and 13% in the MyoF^Rich^ BCAAT subtype. It is evident that 60% of TNBC cases had a vascular type of BCAAT, while the rest had a fibroblast-rich BCAAT subtype. For the TNBC subgroup, BMI and age were inversely correlated to survival (*p* = 0.019 and *p* = 0.024, respectively, Table 2 and Figure 2).

Regarding the relationship between neoadjuvant therapy and clinical–pathological parameters, we found a significant correlation between Epirubycin/Cyclophosphamide (EC) treatment and months of survival (*p* = 0.011), as well as between the V^Rich^ BCAAT and VI^Rich^ BCAAT subtypes and EC therapy (*p* = 0.02). When we analyzed the impact of taxanes on TNBC cases (for cases where this adjuvant therapy was recommended), we found that docetaxel (DTX) had no impact on survival for non-BCAAT subtypes, but paclitaxel (PTX) had a significant correlation to survival (*p* = 0.006). Thus, we concluded that for the TNBC subgroup, 4EC + 12xPTX is more effective than 4xEC + 4xDTX therapy in relation to survival. The next step of our research was to identify the impact of adjuvant therapies used for the TNBC subgroup. Two types of adjuvant therapies were considered: letrozole and capecitabine. We did not have enough cases to evaluate capecitabine’s impact on survival or BCAAT subtype but, for letrozole therapy, we found a significant correlation with months of survival and overall survival (*p* = 0.022 and *p* = 0.035, respectively). No other correlations related to BCAAT subtype or TLS presence were identified for the TNBC molecular subtypes.

The Luminal B (LB) subgroup (19 out of a total of 53 cases included, 35.84%) was the next BC molecular subtype analyzed. The results showed that 26% of LB cases were associated with the F^Rich^ BCAAT subtype, 42% the V^Rich^ BCAAT subtype, and 32% the VI^Rich^ BCAAT subtype. As for the previous group, we analyzed the interrelation of tissular factors (TLS, BCAAT subtype) with clinical parameters (age, BMI), neoadjuvant/adjuvant therapy, and survival.

The LB molecular subtype was the most complex group regarding significant correlations between all the parameters mentioned above. We can assume that the LB subgroup is characterized by the inflammatory and vascular tumor microenvironment, which has a strong impact on therapy response and survival. BMI was directly correlated to the presence of tertiary lymphoid structures (TLSs) (*p* = 0.027, Table 3), supporting the theory that the inflammatory status of the tumor microenvironment is increased in overweight and/or obese BC patients compared to normal-weight BC patients.

Neoadjuvant and adjuvant therapies were also analyzed for this subgroup. For the Epirubycin/Cyclophosphamide (EC) combination, our data support an inverse correlation to BCAAT subtype (*p* = 0.020). The same was found for docetaxel (DTX) (*p* = 0.017). Given that the most predominant BCAAT subtypes were identified as the V^Rich^ and VI^Rich^ BCAAT subtypes; these inverse correlations support that the combination of EC and DTX may favor an F^Rich^ BCAAT subtype containing a high quantity of cancer-associated fibroblasts (CAFs), which are known to be a prognostic marker of worse survival. This finding was also supported by our result of a significant inverse correlation between BCAAT subtype and survival (*p* = 0.05) (Table 4).

When we compared the association of PTX versus DTX with EC, we found that the use of PTX was positively correlated to disease-free survival (DFS, *p* = 0.014, Table 5) but not with overall survival (OS), while DTX did not show any significant correlation to survival.

For the Luminal A (LA) subgroup, 45% of the cases were associated with the V^Rich^ BCAAT subtype, 33% with the VI^Rich^ BCAAT subtype, 11% with the F^Rich^ BCAAT subtype, and 11% with the Myo F^Rich^ BCAAT subtype.

In a global analysis of LA cases, the overall survival (OS) but not disease-free survival (DFS) was highly correlated to some therapies for this BC subtype independent of the BCAAT subtype.

OS was significantly positive and influenced by therapy with DTX and HER/PER (*p*< 0.001 and *p* < 0.001, respectively). For other therapies, we did not detect any significant interrelation with survival and/or BCAAT subtype (Table 6).

Due to the predominance of vascular subtypes of BCAAT, we analyzed these two subgroups separately. The previous findings were confirmed. Additionally, we found that paclitaxel (PTX) therapy was inversely correlated to V^Rich^ and VI^Rich^ BCAAT subtypes, suggesting that PTX therapy may induce fibroblast transformation in BCAAT (Table 7).

Due to our findings regarding the impact of BCAAT subtypes on chemotherapy response, we analyzed the survival rate of patients with different molecular subtypes. We also examined survival rate differences between various therapeutic regimens and BCAAT subgroups.

**Figure 3:**
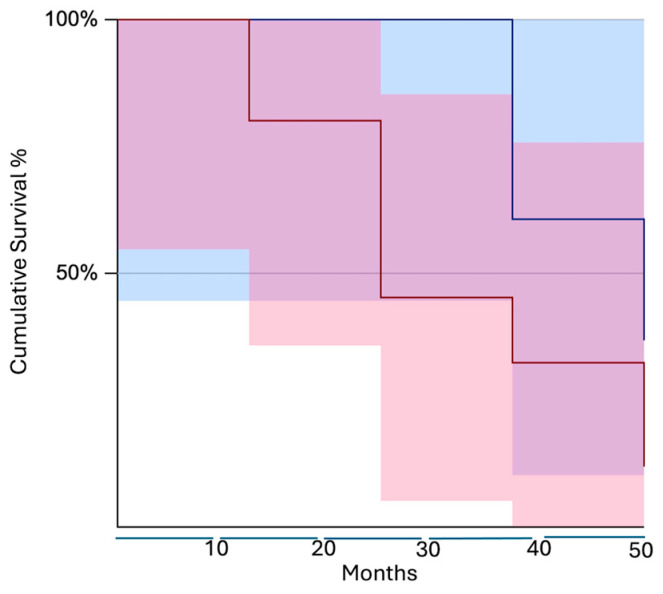
Comparative Kaplan–Meier survival curve for disease-free survival (DFS) between patients receiving the EC + PTX regimen and having associated V^Rich^ and VI^Rich^ BCAAT subtypes (blue curve) compared to the F^Rich^ BCAAT subgroup (red line) receiving similar therapy (log-rank test z = 1.99; *p* = 0.0465).

The TNBC subgroup was divided according to BCAAT subtypes and EC + PTX therapy. More than 75% of TNBC cases were associated with the F^Rich^ BCAAT subtype, and 25% of TNBC cases were associated V^Rich^ and VI^Rich^ BCAAT subtypes. We observed a significant decrease in disease-free survival in the EC + PTX-treated F^Rich^ BCAAT subgroup (Figure 3, red line) compared to the V^Rich^ and VI^Rich^ BCAAT subgroups, which received similar therapy (Figure 3, blue line).

**Figure 4:**
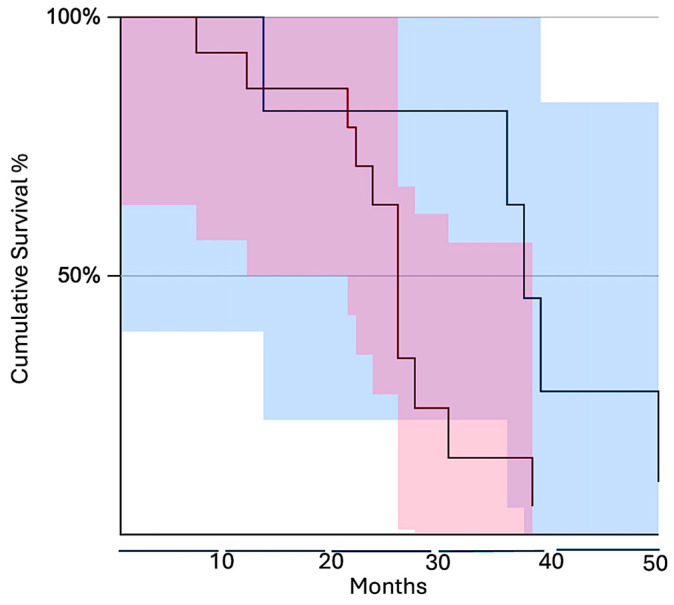
Comparative Kaplan–Meier survival curve for disease-free survival (DFS) between LB patients receiving the EC + PTX regimen and those associated with V^Rich^ and VI^Rich^ BCAAT subtypes (blue curve) compared to the subgroup that did not receive this combination, including the F^Rich^ BCAAT subtype (red line) in a percentage (35.72%) (log-rank test z = 2.22, *p* = 0.0265).

We observed that the V^Rich^ BCAAT subtype was mostly associated with the LB subgroup treated with EC + PTX. For this subgroup, only one case was associated with the VI^Rich^ BCAAT subtype. Comparatively, the V^Rich^ and VI^Rich^ BCAAT subtypes were detected in 64.28% of the LB subgroup not treated with the EC + PTX regimen. For the same subgroup, 35.72% of cases were associated with F^Rich^ BCAAT, with a direct impact on survival rate. As shown in Figure 4, the group that included the F^Rich^ BCAAT subtype had a significant lower survival rate (red line) compared to the V^Rich^ and VI^Rich^ BCAAT subgroups (blue line).

The Luminal B-HER2 (LB-HER2) subgroup comprised a small number of cases. Despite this weakness, we decided to evaluate this subgroup due to its importance and controversial nature. Our group included only seven LB-HER2 cases (13.2% of all cases). For the LB-HER2 subgroup, PTX use was inversely correlated to TLSs (*p* = 0.017). A similar finding was detected for therapy with Herceptin and/or Pertuzumab (HER/PER) (*p* = 0.031). Statistical analysis was performed with above results but, due to the extremely small sample size < 10), we decided to repeat the analysis when the LB-HER2 patients’ cohort increases. For now, we only report data as preliminary findings without any discussions related to this subgroup.

For the HER2 subgroup, due to the extremely small sample size (n = 3), we did not perform statistical analysis.

**Figure 5:**
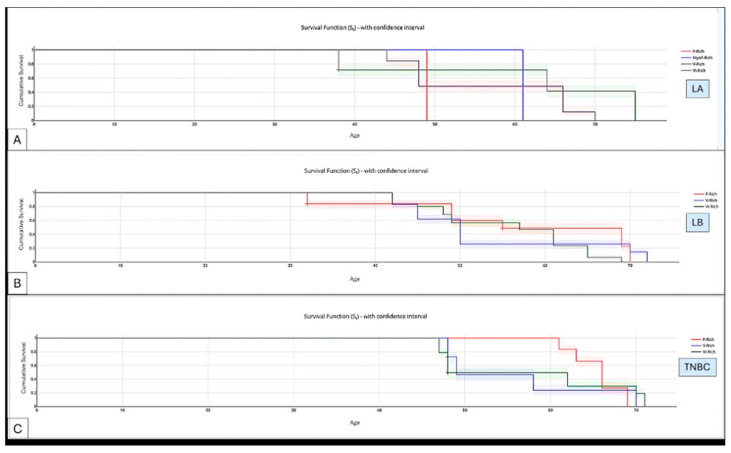
Comparative view of Kaplan–Meier survival curve for Luminal A (LA), Luminal B (LB), and triple-negative breast cancer (TNBC) related to age and BCAAT subtypes. For LA (**A**), the *p*-value equals 1.11022 × 10^−16^, (*p*(x ≤ χ^2^) = 1.00000), meaning that the chance of type1 error is small: 1.110 × 10^−16^ (1.1 × 10^−14^%). The test priori power is strong: 0.9969. For LB (**B**), the *p*-value equals 0.000313924, (*p*(x ≤ χ^2^) = 0.999686), meaning that the chance of type1 error is small: 0.0003139 (0.031%). The test priori power is strong: 1.000. The *p*-value for TNBC (**C**) equals 0.0241859, (*p*(x ≤ χ^2^) = 0.975814), meaning that the chance of type1 error is small: 0.02419 (2.42%). The test priori power is strong: 0.9999.

The integration of other clinico-pathologic factors followed the previous analyses. We assessed BCAATs correlated with multiple factors (BMI, tumor stage, age, therapy) for each BC molecular subtype, which we present here in a comparative manner. When we analyzed the impact of BCAAT on survival related to age, we found that the curves are comparable at first but start to diverge between the ages of 45 and 55. The F^Rich^BCAAT and MyoF^Rich^ BCAAT curves decline more abruptly and at an earlier stage, indicating poorer survival rates in intermediate-to-senior age groups. Patients with V^Rich^ BCAAT appeared to sustain the highest survival rates into their late 50s and 60s before experiencing a decline. The VI^Rich^ BCAAT group is positioned between the superior and inferior groups, with reductions in the range of 50–70. V^Rich^ BCAAT seems to have the best survival profile in LA, whereas F^Rich^BCAAT and MyoF^Rich^ BCAAT appear to have the worst. The groupings diverge most distinctly within the 50–70 age range (see Figure 5A).

Compared to the LA BC subtype, for the LB BC subtype, all groups exhibit a more progressive decline. The F^Rich^ BCAAT, V^Rich^ BCAAT, and VI^Rich^ BCAAT curves are fairly similar. Most of the decline happens between the ages of 50 and 70; there is no significant early separation. Although the changes are small, the F^Rich^ BCAAT group may have a slightly better survival rate in the 50–60-year range, but survival probability for this subgroup is the earliest to decline. The BCAAT subtypes do not differ significantly in LB cancers related to an age below 50. After the age of about 55, there is very little difference in survival patterns across the groups (Figure 5B).

For the TNBC BC subtype, significant declines are observed between the ages of 50 and 60. The F^Rich^ BCAAT group demonstrates sustained performance, exhibiting minimal early declines. V^Rich^ BCAAT and VI^Rich^ BCAAT cluster together and diminish earlier. The disparity between F^Rich^ BCAAT and the other groups becomes increasingly apparent between the ages of 55 and 65. Once more, the curves align around age 70. In the context of TNBC, F^Rich^ BCAAT is associated with improved survival outcomes, whereas V^Rich^ BCAAT and VI^Rich^ BCAAT show less favorable results (see Figure 5C).

**Figure 6:**
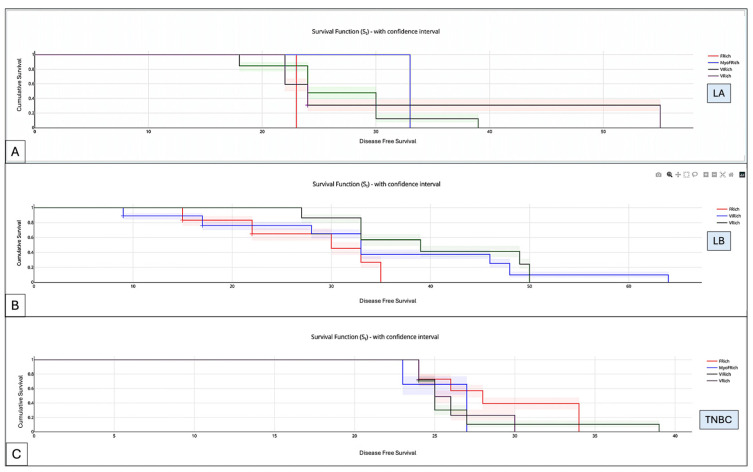
BMI’s influence on BC patients’ survival, assessed separately according to molecular subtypes ((**A**)—LA; (**B**)—LB; (**C**)—TNBC) and BCAAT subgroups. For LA, the *p*-value equals 2.22045 × 10^−15^, (*p*(x ≤ χ^2^) = 1.00000), meaning that the chance of type1 error is small: 2.220 × 10^−15^ (2.2 × 10^−13^%). The test priori power is strong: 0.9915. For LB, the *p*-value equals 0.00000, (*p*(x ≤ χ^2^) = 1.000000), meaning that the chance of type1 error is small: 0.000 (0.0%). The test priori power is strong: 1.000. For TNBC, the *p*-value equals 0.00000119361, (*p*(x ≤ χ^2^) = 0.999999), meaning that the chance of type1 error is small: 0.000001194 (0.00012%). The test priori power is strong: 1.000.

In Figure 6A–C, each panel presents a comparison of disease-free survival (DFS) for LA, LB, and TNBC across four distinct BCAAT subtypes related to BMI. All four BCAAT subgroups maintained a survival rate close to 1.0 until approximately 15 to 20 months when analyzed in relation to BMI. Subsequently, all groups exhibit a significant decline; no group demonstrates consistent superiority. The F^Rich^ BCAAT group seems to exhibit a marginally higher survival rate in the later stages of follow-up; however, the confidence intervals show significant overlap. There is no distinct, clinically significant differentiation between the groups; the LA subtype demonstrates comparable DFS across the groups when it is correlated to BMI (Figure 6A). Compared to LA, there are more events and slower decreases. The decrease tends to happen earlier for the F^Rich^ BCAAT (red curve, Figure 6B), suggesting slightly poorer DFS. The V^Rich^ BCAAT and VI^Rich^ BCAAT curves’ tracks are tightly aligned in the center. We may interpret this as some divergence, for F^Rich^ BCAAT performs somewhat worse and VI^Rich^ BCAAT somewhat better, but the differences still appear modest and are likely not statistically strong.

For TNBC, up to about 20 to 23 months, all four curves stay at 1.0; beyond that, there are significant drops (Figure 6C). All curves decline to poor survival rates by approximately 35 to 40 months. The four groups are not consistently arranged, and they mostly overlap. TNBC exhibits consistently unfavorable disease-free survival, with no discernible distinctions across BCAAT subgroups when it is analyzed in relation to BMI. The trends indicate that BMI does not significantly differentiate DFS among various breast cancer subtypes related to BCAAT subtypes, except for potentially minor variations in LB. All data indicate no or minimal impact of BMI related to survival stratified by BCAAT subgroups.

The next step in our study was to determine if there are any survival differences between the BC molecular subtypes (LA, LB, and TNBC) depending on the therapies associated with the BCAAT subtypes (we chose V^Rich^ BCAAT and VI^Rich^ BCAAT due to their predominance in all three BC subtypes). For the LA subtype, we assessed the impact of PTX and letrozole, while for LB and TNBC, we focused on survival differences between PTX and DTX use correlated to BCAAT subtype.

**Figure 7:**
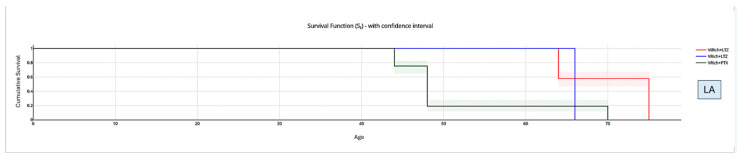
Differences in survival for LA molecular subtype related to BCAAT subtypes associated with letrozole (LTZ) or paclitaxel (PTX) therapy. The *p*-value equals −2.22045 × 10^−16^, (*p*(x ≤ χ^2^) = 1.000000). It means that the chance of type1 error is small: −2.220 × 10^−16^ (−2.2 × 10^−14^%). The test priori power is strong: 0.9878.

Figure 7 presents a comparison of the impact of letrozol (LTZ) and paclitaxel (PTX) associated with V^Rich^ BCAAT and VI^Rich^ BCAAT for the LA_BC subtype. Until about age 65 to 70, the V^Rich^ BCAAT + LTZ subgroup (blue curve) maintains a cumulative survival of 1.0 before abruptly declining. This implies that the drop was caused by a single event and that no events were seen in this group until subsequent ages. The VI^Rich^ BCAAT + LTZ (red curve) remains high until roughly age 55–60, at which point it displays one decline, followed by another at around age 70+, suggesting several events dispersed over time and a lower overall survival rate than the V^Rich^ BCAAT + LTZ subgroup. The V^Rich^ BCAAT + PTX (green curve) has the lowest survival trajectory of the three, dropping first in the mid-40s and then again around age 70.

**Figure 8:**
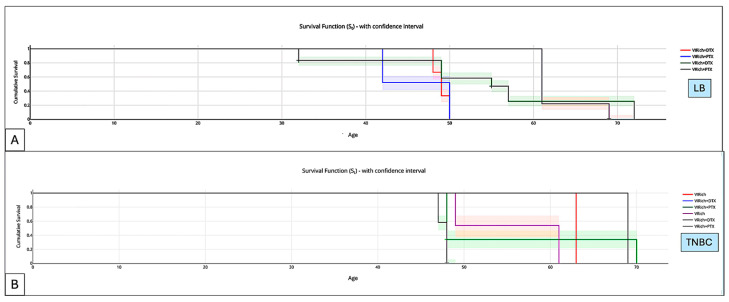
Kaplan–Meier survival curves for LB and TNBC molecular subtypes evaluated by association between the V^Rich^ BCAAT and VI^Rich^ BCAAT subgroups with PTX and DTX therapy. For LB (**A**), the *p*-value equals 1.11022 × 10^−15^ (*p*(x ≤ χ^2^) = 1.00000), meaning that the chance of type1 error is small: 1.110 × 10^−15^ (1.1 × 10^−13^%). The test priori power is strong: 0.9998. For TNBC (**B**), the *p*-value equals 1.22125 × 10^−15^, (*p*(x ≤ χ^2^) = 1.00000), meaning that the chance of type1 error is small: 1.221 × 10^−15^ (1.2 × 10^−13^%). The test priori power is strong: 0.9809.

For the LB and TNBC molecular subtypes (Figure 8A,B), interesting differences were observed. For the LB subgroup, the cases with VI^Rich^ BCAAT had the shortest survival for both DTX and PTX therapy, while the V^Rich^ BCAAT patients showed the longest survival (individuals treated with DTX had longer survival than those treated with PTX despite the occurrence of several events and their early appearance; Figure 8A).

TNBC cases with V^Rich^ BCAAT treated with DTX had the shortest survival rate, shorter than non-treated V^Rich^ BCAAT and VI^Rich^ BCAAT subgroups. The V^Rich^ BCAAT + PTX and VI^Rich^ BCAAT + PTX subgroups showed the longest survival rate (Figure 8B).

F^Rich^ BCAAT is rare among BC molecular subtypes, with a slight increase in prevalence in TNBC and LB. For this reason, we decide to assess it comparatively between BC molecular subtypes.

**Figure 9:**
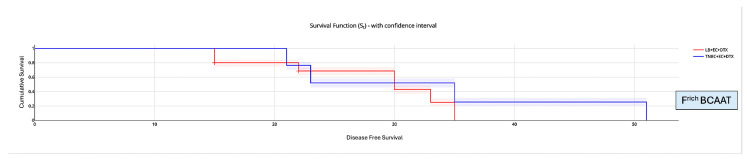
Comparative assessment of survival for F^Rich^ BCAAT subgroup across LB and TNBC treated with DTX. The *p*-value equals 8.56451 × 10^−8^, (*p*(x ≤ χ^2^) = 1.00000), indicating that the chance of type1 error is small: 8.565 × 10^−8^ (0.0000086%). The test priori power is strong: 1.000.

As shown in Figure 9, the F^Rich^ BCAAT _LB subtype group treated with DTX and letrozol (LTZ) had a lower survival rate compared to a similar TNBC subgroup receiving similar therapy. It seems that DTX therapy is the most impactful in relation to the increase in DFS in the F^Rich^ BCAAT_TNBC subgroup.

The next step was to perform multivariate analysis of covariance (MANCOVA).

For this, we analyzed the PTX effects on survival depending on BCAAT subtypes related to age and BMI globally for all cases included in the study. The results are summarized in Table 8.

After accounting for age and body mass index (BMI), the MANCOVA revealed a significant interaction effect between PTX and BCAAT on survival rates (*p* = 0.008). This suggests that the impact of PTX on survival rates varies between BCAAT subgroups. Covariate AGE was also statistically significant (*p* = 0.006).

We then performed the same analysis for each BC molecular subtype to identify the potential impact of PTX therapy on survival related to BCAAT subtype, age, and BMI.

For the LA BC molecular subtype, we had an extremely low number of cases but were still able to carry out a MANCOVA. No significant impact on survival was found for PTX therapy related to BCAAT subgroup.

MANCOVA was performed to investigate the effects of PTX therapy, BCAAT subgroup, age, and body mass index (BMI), as well as the interaction between these factors, on disease-free survival (DFS) and overall survival (OS) for the LB BC molecular subtype. PTX had a multivariate effect that was borderline significant (Wilks’s Lambda = 0.606, F(2,11) = 3.58, *p* = 0.064), and the BCAAT subgroup had a significant effect (Wilks’s Lambda = 0.402, F(4,22) = 3.18, *p* = 0.033). Both of these findings were based on statistical analysis. PTX × BCAAT interaction, age, and body mass index (BMI) did not have a significant impact (Table 9).

It was found that PTX was substantially linked to better survival when it was subjected to univariate analysis for DFS (F(1,12) = 5.07, *p* = 0.044). There was a statistically significant association between the BCAAT subgroup and the outcome for OS (F(2,12) = 6.68, *p* = 0.011). It was discovered that there were no significant interactions between the PTX and BCAAT subgroups for either DFS or OS (Table 10 and Table 11).

For the TNBC BC molecular subtype, PTX therapy seems to have a strong impact on survival depending on and differentiated by BCAAT subgroups.

MANCOVA results for the TNBC subgroup revealed a significant *p* value for BCAAT subgroups (*p* = 0.045), as shown in Table 12.

Thus, we performed a multivariate regression analysis to examine the effect of PTX on disease-free survival (DFS), controlling for age and body mass index (BMI), across three out of four different BCAAT subgroups (F^Rich^, VI^Rich^, and V^Rich^). The improvement in clinical outcomes was substantially linked with PTX, according to the results (β = 0.50, *p* = 0.03).

When the data were stratified by BCAAT subgroups, patients with VI^Rich^ BCAAT had better disease-free survival than those with F^Rich^ BCAAT (β = 0.30, *p* = 0.04). However, there was no statistically significant difference between V^Rich^ BCAAT and F^Rich^ BCAAT (β = 0.10, *p* = 0.40). The results showed that age was a strong negative predictor of outcome (β = −0.02, *p* = 0.05), suggesting that the benefit of PTX decreases with age. On the other hand, BMI was not a separate predictor (β = −0.01, *p* = 0.50, Table 13).

Most importantly, interaction effects showed that patients with VI^Rich^ BCAAT benefited the most from PTX as a therapeutic agent (PTX × VI^Rich^ BCAAT: β = 0.45, *p* = 0.02). Further, there were significant negative interactions for PTX × Age (β = −0.03, *p* = 0.04) and PTX × BMI (β = −0.04, *p* = 0.04), indicating that the effectiveness of PTX was reduced in older and higher-BMI individuals (see Table 13).

**Figure 10:**
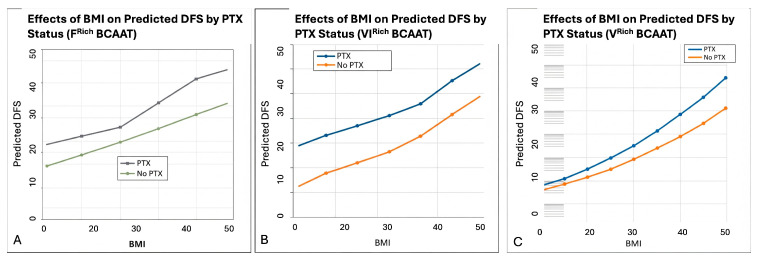
Mutual influences of BMI and BCAAT subgroup related to PTX therapy response and predicted disease-free survival (DFS). It has been shown that for the VI^Rich^ BCAAT subtype, PTX has a significant impact on DFS compared to the non-treated group (**B**). For the F^Rich^ (**A**) and V^Rich^ (**C**) BCAAT subtypes, DFS is slightly but not significantly increased by PTX therapy. All are highly influenced by BMI.

Charts related comparing BCAAT subgroups, PTX, and BMI are shown in Figure 10.

**Figure 11:**
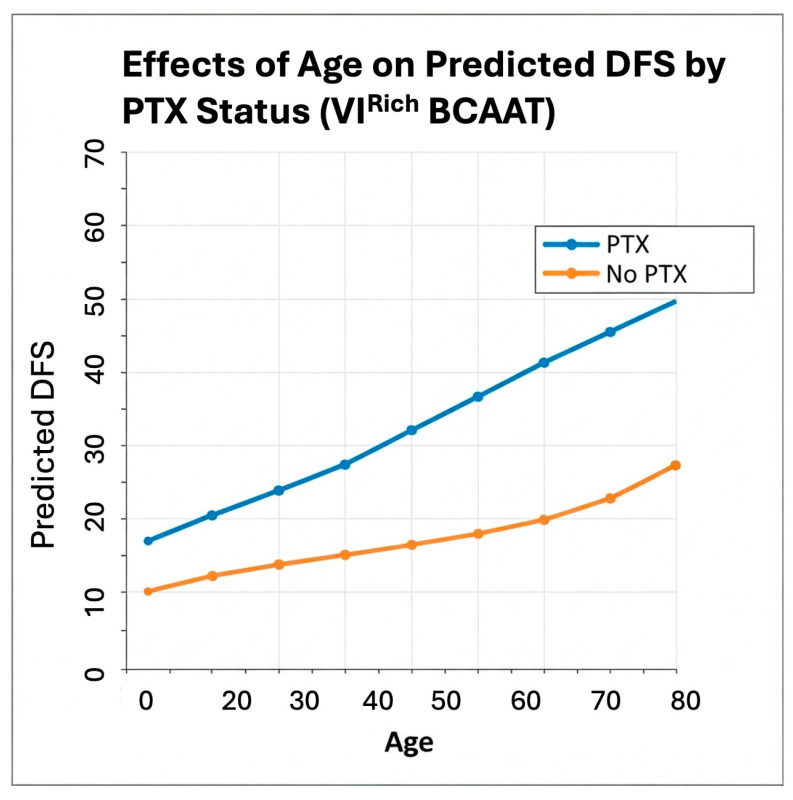
Influence of age on BCAAT VI^Rich^ subgroup related to PTX and DFS. Predicted DFS (y-axis) is expressed in months and age (x-axis) expressed in years.

Age has an impact on the VI^Rich^ BCAAT subgroup, depending on PTX therapy (Figure 11).

Taken together, our results show that PTX responsiveness is patient-specific and depends on the BCAAT subgroup. Younger people with a lower body mass index, especially those in the VI^Rich^ BCAAT group, showed the most improvement. Aligning with the outcomes of current precision medicine techniques, these data provide support for incorporating patient demographics and molecular categorization into the clinical decision-making process for PTX.

## 4. Discussion

It is now known that the adipose microenvironment, in addition to intrinsic tumor features, shapes the course of breast cancer. The molecular importance of adipose tissue in breast cancer requires further investigation as obesity rates rise globally. By secreting hormones, cytokines, fatty acids, and other mediators, adipose tissue acts as an active endocrine organ that can control tumor activity. According to the research, in response to nearby cancer cells, cancer-associated adipocytes (CAAs) undergo metabolic and phenotypic reprogramming. CAASs release substances that may promote tumor growth, invasion, and metastatic potential [38,39,40]. These findings support a more general theory that different cellular states in peritumoral adipose tissue can affect long-term prognosis and therapeutic response.

Based on cellular, inflammatory, and vascular characteristics, our previous research revealed four histologically characterized subgroups of breast cancer-associated adipose tissue (BCAAT) [29]. High concentrations of CD34- and SMA-positive fibroblast-lineage cells are characteristic of the F^Rich^/MyoF^Rich^ subtype. Patients between the ages of 35 and 49 were more likely to have this subtype, which was linked to worse survival rates. Building on previous results, we reported that patients with TNBC_F^Rich^_BCAAT treated with EC + docetaxel (DTX) had a higher survival rate than LB_F^Rich^ BCAAT patients treated with EC + docetaxel (DTX). The idea that DTX may interact with the F^Rich^BCAAT microenvironment is supported by this pattern. 

Bidirectional communication between cancer cells and adipocytes appears to be crucial in determining malignant activity outside of fibroblast-rich settings. Inflammatory mediators and pro-tumorigenic adipokines are released by obese adipocytes, which may encourage aggressive tumor morphologies [41]. Moreover, cancer cell migration, immune cell recruitment, chronic inflammation, and chemoresistance to drugs like doxorubicin and docetaxel have all been linked to adipocyte-derived exosomes carrying microRNA and other cargo [42,43,44,45]. However, the existence of fibroblasts and myofibroblasts within peritumoral adipose tissue has not been taken into consideration in most previous investigations, leaving questions that BCAAT subtyping may help resolve.

Additionally, vascular-rich BCAAT subtypes may have distinct effects on treatment response, according to the current data. Epirubicin/cyclophosphamide (EC) treatment was associated with better disease-free survival in V^Rich^ and VI^Rich^ BCAAT in TNBC cases. This indicates that higher vascular density may enable better drug penetration into the peritumoral adipose compartment. EC’s proven anti-angiogenic effect [46,47] may nevertheless interact with a vascular-rich milieu to promote advantageous drug distribution profiles, especially in TNBC, even though it was not administered metronomically.

The connection between BCAAT and paclitaxel seems especially significant. Although paclitaxel is known to exert anti-angiogenic effects by suppressing VEGF [48], it may also encourage vascular normalization by lowering inflammatory signals and stabilizing the endothelial architecture. This normalization may improve therapeutic delivery and perfusion to nearby adipose compartments, as well as malignant tissue. PTX was associated with higher overall survival in TNBC and better disease-free survival in LB breast cancer in our population, primarily in the V^Rich^ and VI^Rich^ BCAAT subtypes. These findings support the theory that vascular-normalized BCAAT improves chemotherapeutic efficacy and are consistent with growing evidence that PTX may increase drug distribution in adipose-rich microenvironments [49,50].

Regardless of BCAAT subtype, the PTX and HER/PER treatments showed an inverse relationship with tertiary lymphoid structures (TLSs) in the LB-HER2 subgroup. According to reports for HER2-positive illness, this observation might be the result of therapy-induced lymphocyte mobilization [51,52,53,54]. This mobilization highlights the intricate relationships between therapy, local immunity, and adipose microenvironments, even if it did not correlate with improved OS or DFS in our sample.

When combined, these results provide credence to the general theory that therapeutic response and survival are strongly influenced by heterogeneity within adipose tissue associated with breast cancer. V^Rich^ BCAAT may increase the efficacy of EC and PTX via improved perfusion and drug delivery. F^Rich^ BCAAT may contribute to DTX-associated effects in some but not all BC molecular subtypes. Therefore, incorporating BCAAT profiling into clinical evaluation may offer fresh perspectives on treatment stratification and uncover microenvironmental mechanisms that call for additional mechanistic research.

As we previously stated, this was an exploratory study. Exploratory research studies often employ modest sample sizes, usually between 20 and 150 participants. These investigations prioritize identifying patterns, generating hypotheses, or exploring novel phenomena over providing convincing proof, emphasizing flexibility and depth instead of extensive statistical power.

In examining the three molecular subtypes related to the impact of age and BCAAT subgroups, the survival curves indicated that individuals with different BCAATs and obesity generally exhibit diminished age-adjusted survival rates, with the distinction being particularly evident in TNBC. This pattern aligns with extensive cohort data indicating that the invasion of adipose tissue is independently linked to poorer overall survival outcomes in breast cancer, particularly in TNBC [55]. In the LA subtype, the curves start to separate during middle age. Groups associated with BCAATs showed a tendency toward reduced cumulative survival rates. This is consistent with findings that adipose invasion enhances inflammatory signaling and plays a role in endocrine resistance, thereby exacerbating outcomes even in hormone-receptor-positive conditions [55]. In LB, the overall survival pattern exhibits a more constrained nature. However, the groups associated with adipose tissue continue to display a nuanced decline. LB cancers, characterized by elevated proliferation rates compared to LA, may exhibit heightened sensitivity to adipocyte-derived cytokines like IL-6 and leptin. These may contribute to increased tumor aggressiveness and metabolic support. These mechanisms have been consistently documented in co-culture and in vivo models [56].

The most notable trend is observed in TNBC, which is characterized by a more pronounced and earlier decline in adipose-associated curves. In TNBC, cancer-associated adipocytes facilitate progression through mechanisms such as lipid transfer, CCL5 expression, IL-6 secretion, and the activation of STAT3/NF-κB pathways. The mechanisms driven by adipose tissue exhibit a significant correlation with metastasis and a diminished overall survival rate in TNBC [57,58].

Adipose tissue plays an active, not passive, role. The presence of this factor—which manifests through direct adipose invasion, adipocyte-rich stroma, or metabolic programming associated with adipogenesis—exhibits a correlation with diminished survival across various subtypes, displaying a mild effect in LA, a moderate impact in LB, and a notably pronounced influence in TNBC. The results advocate for a broader exploration of interventions aimed at the microenvironment or metabolic pathways, especially concerning TNBC associated with adipose tissue.

The presence of F^Rich^ BCAAT typically indicates a microenvironment characterized by high stroma and pro-tumorigenic properties, which carries particular significance for therapeutic responses in TNBC [59].

The presence of fibroblast-rich stroma correlates with enhanced extracellular matrix remodeling and elevated proliferative pressure, frequently resulting in increased sensitivity to microtubule-targeting agents. This corresponds with our insight that DTX serves as the main driver enhancing DFS within the F^Rich^ BCAAT -TNBC subgroup [60,61].

This study’s primary strengths are as follows: (1) it provides preliminary evidence of a differential, specific impact of BCAAT subtypes on therapy response and prognosis of BC patients; (2) identification of the V^Rich^ and VI^Rich^ BCAAT subgroups as key players in the response to therapy in relation to survival for patients with TNBC and LB BC molecular subtypes; (3) evidence supporting the choice of PTX and the association of EC + PTX as a therapeutic choice for TNBC and LB molecular subtypes related to BCAAT subtypes (for V^Rich^ and VI^Rich^ BCAAT), which contribute to patient survival; (4) the finding that docetaxel (DTX) may have a controversial impact depending on BCAAT subgroup, with a variable impact on prognosis and survival. The main weakness of this study could be considered its small sample size (n = 53); however, given its exploratory nature, a sample size between 20 and 150 is acceptable. This small sample size was the result of limiting the case selection to a single oncology center (Oncohelp Hospital Timisoara), which may be considered another weakness of the current study. The single-center analysis presented in this paper can be shared with people abroad, outside our own experience, and concerns cases from western Romania. The results of this study may be considered the basis of a hypothesis, which must be validated in large cohorts.

## 5. Conclusions

The effects of BCAAT subtypes are not uniform; rather, they are graded, with low-severity effects in LA, intermediate-severity effects in LB, and the most severe effects in TNBC. The response to neoadjuvant therapy and the survival of patients with breast cancer may be affected by not only the molecular subtype of breast cancer but also the BCAAT subgroup, independent of body mass index. In particular, the TNBC and LB molecular subtypes could be linked to V^Rich^ and VI^Rich^ BCAAT profiles, which demonstrate a notable responsiveness to EC + PTX therapy, potentially resulting in enhanced overall survival and disease-free survival. In the context of the LA subtype, the combination of EC and DTX may emerge as the most efficacious neoadjuvant strategy. It is imperative that these relationships undergo thorough examination and validation within more extensive cohort studies.

## Figures and Tables

**Table 1:** Patients’ data relating to the aim of the study. The following information is provided for each patient: body mass index (BMI), age, months of survival, BCAAT subtype (*F^Rich^*—fibroblast rich; *MyoF^Rich^*—myofibroblast rich; *V^Rich^*—vascular rich; *VI^Rich^*—mixed vascular/inflammatory rich [29], tertiary lymphoid structures ((TLS), 0—absence of TLS; 1—presence of TLSs), molecular subtypes (TNBC-triple negative breast cancer; LB-HER+—Luminal B/HER; LB—Luminal B; LA—Luminal A; HER2—Human Epidermal Receptor 2-positive), neoadjuvant therapy (EC—Epirubicin/Ciclofosfamid; ECdd—Epirubicin/Ciclofosfamide dense dose; DTX—docetaxel; PTX—paclitaxel.

No. of Patient	BMI	Age	Disease-Free Survival (Months)	BCAAT Subtype	Tumor Grade	Stage	TLS	Neoadjuvant Therapy	Molecular Subtype
1	25.59	49	33.00	*VI^Rich^*	3	IIA	0	4xEC, 4xDTX	TNBC
2	27.85	71	31.00	*V^Rich^*	3	IIB	1	4xEC + 4xDTX	TNBC
3	39.11	70	17.00	*V^Rich^*	3	IIIA	1	4xEC + 12xPTX	TNBC
4	25.71	70	30.00	*VI^Rich^*	3	IIA	1	4xEC, 4xDTX	TNBC
5	26.45	69	35.00	*MyoF^Rich^*	2	IIIB	1	4xEC, 4xDTX	TNBC
6	26.45	69	35.00	*F^Rich^*	2	IIIA	1	4xEC, 4xDTX	TNBC
7	24.01	66	51.00	*F^Rich^*	2	IIB	0	4xEC	TNBC
8	34.85	63	23.00	*F^Rich^*	2	IIA	1	0	TNBC
9	27.34	62	32.00	*V^Rich^*	2	IIA	0	4xEC, 4xPTX + 4xHER/PER	TNBC
10	28.62	61	21.00	*F^Rich^*	2	IIA	0	0	TNBC
11	30.01	58	29.00	*VI^Rich^*	2	IIA	1	4xEC,4xPTX	TNBC
12	23.05	49	18	*MyoF^Rich^*	2	IIB	1	8xPT	TNBC
13	24.3	48	35.00	*VI^Rich^*	2	IIA	1	4ECdd + 12xPTX, RTE	TNBC
14	24.3	48	48.00	*V^Rich^*	3	IIIA	0	4ECdd + 12xPTX, RTE	TNBC
15	25.34	47	35.00	*V^Rich^*	3	IIIA	1	4EC-dd + 12xPTXw	TNBC
16	25.39	64	33.00	*VI^Rich^*	3	IIA	1	4xEC-dd + 4xPTX	LB-HER+
17	30	63	0.00	*V^Rich^*	3	IIB	1	6xEC	LB-HER+
18	27.34	62	32.00	*V^Rich^*	2	IIA	0	4xEC, 4xPTX + 4xHER/PER	LB-HER+
19	29.82	61	64.00	*V^Rich^*	2	IIA	0	4xEC-dd + 12xPTX + trastuzumab	LB-HER+
20	36.65	57	48	*MyoF^Rich^*	2	IIB	1	0	LB-HER+
21	26.35	38	25.00	*F^Rich^*	2	IIIB	1	4xEC, 4xDTX + HER/Per	LB-HER+
22	27.14	37	29.00	*F^Rich^*	1	IIIA	1	4xECdd + 4xDTX	LB-HER+
23	25.28	57	28.00	*V^Rich^*	3	IIIB	0	4xEC, 4xDTX + HER/Per	LB
24	27.64	42	46.00	*V^Rich^*	3	IIB	0	4xEC, 12xPTXw	LB
25	22.6	42	39	*VI^Rich^*	2	IIA	1	0	LB
26	29.82	72	33.00	*VI^Rich^*	2	IIIB	1	4xEC, 4xDTX	LB
27	25.71	70	30.00	*F^Rich^*	2	IIA	0	4xEC, 4xDTX	LB
28	25.34	70	27.00	*VI^Rich^*	2	IIA	0	0	LB
29	39.21	69	17.00	*V^Rich^*	2	IIB	1	4xEC, 12xPTXw	LB
30	26.45	69	35.00	*F^Rich^*	2	IIB	1	4xEC, 4xDTX	LB
31	35	65	48.00	*V^Rich^*	2	IIA	0	4xEC, 12xPTXw	LB
32	29.82	61	64.00	*V^Rich^*	2	IIB	1	4xEC-dd + 12xPTX + trastuzumab	LB
33	33.32	55	15	*F^Rich^*	2	IIB	1	4xEC, 4xDTX	LB
34	22.05	50	50.00	*VI^Rich^*	2	IIB	0	4xEC-dd, 12xPTXw + 12xHer/PER	LB
35	23.34	50	33	*VI^Rich^*	2	IIB	0	4XECdd + 6xDTX + CBP	LB
36	25.59	49	33.00	*V^Rich^*	2	IIA	0	4xEC, 4xDTX	LB
37	25.59	49	33.00	*F^Rich^*	2	IIIB	0	4xEC, 4xDTX	LB
38	27.28	48	33.00	*V^Rich^*	2	IIA	1	4EC-DD + 4xDTX	LB
39	36	45	49	*VI^Rich^*	2	IIIB	1	0	LB
40	25.39	45	9.00	*V^Rich^*	1	IA	1	0	LB
41	22.84	32	22.00	*F^Rich^*	2	IIB	0	4xEC,4xDTX + HER/Per + Zoladex	LB
42	39.11	70	17.00	*V^Rich^*	3	IIIC	0	4xEC + 12xPTX	LA
43	23.44	38	36.00	*VI^Rich^*	2	IIB	0	4ECDD + 4XDTX + HER + PER	LA
44	22.31	75	52.00	*VI^Rich^*	2	IIIA	0	0	LA
45	24.01	66	51.00	*V^Rich^*	2	IA	0	4xEC	LA
46	55	64	38.00	*VI^Rich^*	2	IIIB	1	0	LA
47	32.85	61	25.00	*MyoF^Rich^*	2	IIIA	0	4xEC, 12xPTXw	LA
48	23.05	49	18	*F^Rich^*	2	IIIB	0	8xPT	LA
49	29.75	48	50.00	*V^Rich^*	2	IA	1	4xEC, 12xPTXw	LA
50	17.93	44	22.00	*V^Rich^*	2	IA	1	6xEC-DD, 12xPTXw	LA
51	20.07	63	23.00	*VI^Rich^*	2	IIIC	0	4xEC, 4xDTX	HER+
52	33.33	63	27.00	*V^Rich^*	2	IIB	1	0	HER+
53	25.39	64	33.00	*V^Rich^*	2	IIB	1	4xEC-dd + 4xPTX	HER+

**Table 2:** Correlation matrix of clinical–pathological parameters per TNBC subgroup.Significant correlation for * *p* < 0.05.

		BMI	AGE
**Survival**	Pearson’s r	−0.538 *	−0.519 *
	df	15	15
	*p*-value	0.019	0.024
	95% CI Upper	−0.126	−0.099
	95% CI Lower	−1.000	−1.000
	Spearman’s rho	−0.550 *	−0.501 *
	df	15	15
	*p*-value	0.017	0.029
	Kendall’s Tau B	−0.523*	−0.429 *
	*p*-value	0.020	0.030

**Table 3:** Correlation matrix for BMI and TLS for LB subgroup.

		BMI
**TLS**	Pearson’s r	0.456 *
	df	17
	*p*-value	0.050
	95% CI Upper	0.750
	95% CI Lower	−0.007
	Spearman’s rho	0.458 *
	df	17
	*p*-value	0.049
	Kendall’s Tau B	0.434
	*p*-value	0.052

Significant correlation for * *p* < 0.05.

**Table 4:** Correlation matrix of different BCAAT subtypes and neoadjuvant therapies related to survival.

		BCAAT	EC	PTX
**EC**	Pearson’s r	−0.474 *	—	
	df	17	—	
	*p*-value	0.020	—	
	95% CI Upper	−0.104	—	
	95% CI Lower	−1.000	—	
	Spearman’s rho	−0.478 *	—	
	df	17	—	
	*p*-value	0.019	—	
	Kendall’s Tau B	−0.452 *	—	
	*p*-value	0.021	—	
**PTX**	Pearson’s r	0.116	0.309	—
	df	17	17	—
	*p*-value	0.682	0.901	—
	95% CI Upper	0.484	0.623	—
	95% CI Lower	−1.000	−1.000	—
	Spearman’s rho	0.105	0.309	—
	df	17	17	—
	*p*-value	0.665	0.901	—
	Kendall’s Tau B	0.099	0.309	—
	*p*-value	0.672	0.905	—
**DTX**	Pearson’s r	−0.490 *	0.544	−0.630 **
	df	17	17	17
	*p*-value	0.017	0.992	0.002
	95% CI Upper	−0.124	0.770	−0.319
	95% CI Lower	−1.000	−1.000	−1.000
	Spearman’s rho	−0.483 *	0.544	−0.630 **
	df	17	17	17
	*p*-value	0.018	0.992	0.002
	Kendall’s Tau B	−0.456 *	0.544	−0.630 **
	*p*-value	0.020	0.990	0.004
**Survival**	Pearson’s r	−0.476 *	−0.050	−0.015
	df	17	17	17
	*p*-value	0.050	0.419	0.475
	95% CI Upper	0.016	0.346	0.376
	95% CI Lower	−1.000	−1.000	−1.000
	Spearman’s rho	−0.456	−0.050	−0.015
	df	17	17	17
	*p*-value	0.050	0.419	0.475
	Kendall’s Tau B	−0.357	−0.050	−0.015
	*p*-value	0.055	0.416	0.474

Significant correlation for * *p* < 0.05, ** *p* < 0.01.

**Table 5:** Correlation matrix related to survival (disease-free survival—DFS and overall survival (OS) for paclitaxel (PTX) and docetaxel (DTX) related to Luminal B BC molecular subtype.

		Survival (DFS)	PTX	Survival (OS)
**PTX**	Pearson’s r	0.506 *	—	
	Df	19	—	
	*p*-value	0.014	—	
	95% CI Upper	1.000	—	
	95% CI Lower	0.145	—	
	Spearman’s rho	0.462 *	—	
	Df	19	—	
	*p*-value	0.023	—	
	Kendall’s Tau B	0.396 *	—	
	*p*-value	0.025	—	
**Survival** **(OS)**	Pearson’s r	−0.714	−0.015	—
	Df	19	19	—
	*p*-value	1.000	0.525	—
	95% CI Upper	1.000	1.000	—
	95% CI Lower	−0.863	−0.403	—
	Spearman’s rho	−0.713	−0.015	—
	Df	19	19	—
	*p*-value	1.000	0.525	—
	Kendall’s Tau B	−0.610	−0.015	—
	*p*-value	0.999	0.526	—
**DTX**	Pearson’s r	−0.353	−0.630	−0.027
	Df	19	19	19
	*p*-value	0.931	0.998	0.544
	95% CI Upper	1.000	1.000	1.000
	95% CI Lower	−0.653	−0.819	−0.412
	Spearman’s rho	−0.349	−0.630	−0.027
	Df	19	19	19
	*p*-value	0.929	0.998	0.544
	Kendall’s Tau B	−0.299	−0.630	−0.027
	*p*-value	0.931	0.996	0.546

Note: * *p* < 0.05.

**Table 6:** Correlation matrix for the impact of therapy on LA breast cancer survival. Note that neoadjuvant therapy, including Herceptin/pertuzumab (HER/PER) and docetaxel (DTX), significantly improved overall survival but not DFS.

		DFS	Survival	PTX	HER/PER
**PTX**	Pearson’s r	−0.384	−0.316	—	
	df	7	7	—	
	*p*-value	0.846	0.796	—	
	95% CI Upper	1.000	1.000	—	
	95% CI Lower	−0.792	−0.761	—	
	Spearman’s rho	−0.433	−0.316	—	
	df	7	7	—	
	*p*-value	0.878	0.796	—	
	Kendall’s Tau B	−0.373	−0.316	—	
	*p*-value	0.890	0.814	—	
**HER/PER**	Pearson’s r	0.043	1.000 ***	−0.316	—
	df	7	7	7	—
	*p*-value	0.456	<0.001	0.796	—
	95% CI Upper	1.000	1.000	1.000	—
	95% CI Lower	−0.557	1.000	−0.761	—
	Spearman’s rho	0.000	1.000 ***	−0.316	—
	df	7	7	7	—
	*p*-value	0.500	<0.001	0.796	—
	Kendall’s Tau B	0.000	1.000 **	−0.316	—
	*p*-value	0.500	0.002	0.814	—
**DTX**	Pearson’s r	0.043	1.000 ***	−0.316	1.000 ***
	df	7	7	7	7
	*p*-value	0.456	<0.001	0.796	<0.001
	95% CI Upper	1.000	1.000	1.000	1.000
	95% CI Lower	−0.557	1.000	−0.761	1.000
	Spearman’s rho	0.000	1.000 ***	−0.316	1.000 ***
	df	7	7	7	7
	*p*-value	0.500	<0.001	0.796	<0.001
	Kendall’s Tau B	0.000	1.000 **	−0.316	1.000 **
	*p*-value	0.500	0.002	0.814	0.002

Note: Hₐ indicates a positive correlation. ** *p* < 0.01, and *** *p* < 0.001; one-tailed.

**Table 7:** Correlation matrix for vascular subtypes of breast cancer related to paclitaxel (PTX) therapy.

		BCAAT	PTX
**PTX**	Pearson’s r	−0.750 *	—
	Df	5	—
	*p*-value	0.026	—
	95% CI Upper	−0.149	—
	95% CI Lower	−1.000	—
	Spearman’s rho	−0.750 *	—
	Df	5	—
	*p*-value	0.026	—
	Kendall’s Tau B	−0.750 *	—
	*p*-value	0.033	—

Note: Hₐ indicates a negative correlation. * *p* < 0.05, one-tailed.

**Table 8:** MANCOVA of PTX influence on survival depending on BCAAT subtype related to age and BMI. A *p*-value less than 0.05 was considered statistically significant.

Predictor	Wilks’s Lambda	F-Value	df1	df2	*p*-Value
PTX	0.89	1.12	2	49	0.335
BCAAT	0.82	2.15	3	49	0.105
PTX × BCAAT	0.68	3.31	6	49	**0.008**
AGE	0.58	8.22	1	49	**0.006**
BMI	0.93	0.87	1	49	0.356

**Table 9:** Multivariate test statistics (Wilks’s Lambda, MANCOVA) for LB BC molecular subtype. A *p*-value less than 0.05 was considered statistically significant.

Predictor	Wilks’s Lambda	F Value	df1	df2	*p*-Value
PTX	0.6058	3.579	2	11	0.064
BCAAT	0.4018	3.176	4	22	0.033 *
AGE	0.9140	0.517	2	11	0.610
BMI	0.9544	0.263	2	11	0.774
PTX_bin × BCAAT	0.9447	0.322	2	11	0.731

Significant correlation for * *p* < 0.05.

**Table 10:** Univariate ANCOVA for disease-free survival (DFS). A *p*-value less than 0.05 was considered statistically significant.

Predictor	Df	Sum Sq	Mean Sq	F Value	*p*-Value
PTX	1	836.86	836.86	5.07	0.044 *
BCAAT	2	330.25	165.13	1.00	0.396
AGE	1	21.53	21.53	0.13	0.724
BMI	1	36.76	36.76	0.22	0.645
PTX × BCAAT	1	66.23	66.23	0.40	0.538

Significant correlation for * *p* < 0.05.

**Table 11:** Univariate ANCOVA for overall survival (OS).

Predictor	Df	Sum Sq	Mean Sq	F Value	*p*-Value
PTX	1	0.1692	0.1692	1.95	0.188
BCAAT	2	1.1571	0.5786	6.68	0.011 *
AGE	1	0.0924	0.0924	1.07	0.322
BMI	1	0.0355	0.0355	0.41	0.534
PTX × BCAAT	1	0.0327	0.0327	0.38	0.550

Significant correlation for * *p* < 0.05.

**Table 12:** MANCOVA of PTX influence on survival depending on BCAAT, age, and BMI for The TNBC BC molecular subtype. A *p*-value less than 0.05 was considered statistically significant.

Predictor	Wilks’s Lambda	F Value	df1	df2	*p*-Value
**BCAAT**	0.46	2.38	4	18	0.045 *
**PTX**	0.53	1.98	2	9	0.200
**AGE**	0.72	1.01	2	9	0.390
**BMI**	0.81	0.55	2	9	0.590
**BCAAT:PTX**	0.66	1.29	4	18	0.310

Significant correlation for * *p* < 0.05.

**Table 13:** Multivariate regression analysis results demonstrate that PTX was significantly associated with improved patient outcomes highly dependent on BCAAT subgroup, age and BMI for the TNBC BC molecular subtype. A *p*-value less than 0.05 was considered statistically significant.

Predictor	*β*	Std. Error	*p*-Value
**PTX**	0.50	0.20	0.03 *
**BCAAT (VI^Rich^ vs. F^Rich^)**	0.30	0.15	0.04 *
**BCAAT (V^Rich^ vs. F^Rich^)**	0.10	0.12	0.40
**Age**	−0.02	0.01	0.05 *
**BMI**	−0.01	0.02	0.50
**PTX × BCAAT (VI^Rich^)**	0.45	0.18	0.02 *
**PTX × Age**	−0.03	0.01	0.04 *
**PTX × BMI**	−0.04	0.02	0.04 *

Significant correlation for * *p* < 0.05.

## Data Availability

The data presented in this study are available on request from the corresponding author according to Oncohelp Clinic Policies.
